# Gender differences in depressive symptoms of rural Chinese grandparents caring for grandchildren

**DOI:** 10.1186/s12889-021-11886-3

**Published:** 2021-10-11

**Authors:** Dantong Zhao, Zhongliang Zhou, Chi Shen, Sahardid Ibrahim, Yaxin Zhao, Dan Cao, Sha Lai

**Affiliations:** 1 School of Public Policy and Administration, Xi’an Jiaotong University, No. 28 Xianning West Road, Xi’an, 710049 Shaanxi China; 2 School of Public Health, Health Science Center, Xi’an Jiaotong University, Xi’an, China

**Keywords:** Gender differences, Depressive symptoms, Grandchild care, Intensity, Rural China

## Abstract

**Background:**

Caring for grandchildren is regarded as one of the principle roles of middle- and old-aged adults, especially among rural Chinese grandparents. This study aims to examine the gender differences in depressive symptoms of rural Chinese grandparents caring for grandchildren, based on the gender differences in grandparental role engagement and the theories of role strain and role enhancement.

**Methods:**

A total of 4833 rural citizens with one or more grandchildren were selected from the China Health and Retirement Longitudinal Study (CHARLS) conducted in 2015. Grandchild care was measured by continuous variable (duration) and categorical variable (no care, low intensity, moderate intensity, high intensity). Depressive symptoms were assessed by the Center for Epidemiologic Studies Depression Scale (CES-D). We used coarsened exact matching (CEM) to balance the covariates of caregivers and non-caregivers. Following CEM, 1975 non-caregivers and 2212 caregivers were identified (*N* = 4187). Multilevel linear regression was employed to examine the gender differences in depressive symptoms. We also tested for the moderating role of gender on the association between grandchild care and depressive symptoms.

**Results:**

Grandmothers were more likely to provide grandchild care (54.42% vs 51.43%) at high intensity (61.46% vs 51.01%), with longer duration (39.24 h vs 33.15 h) than that given by grandfathers. Grandmothers suffered more from depressive symptoms than grandfathers, and such gap increased when grandparents were involved in high-intensity care. Grandmothers providing grandchild care, particularly at moderate intensity, were associated with fewer depressive symptoms (Coef. = − 0.087, 95%CI: − 0.163, − 0.010; Coef. = − 0.291, 95%CI: − 0.435, − 0.147), compared with non-caregivers. Grandmothers giving moderate intensity of grandchild care were also associated with fewer depressive symptoms (Coef. = − 0.171, 95% CI: − 0.313, − 0.029), compared with those with low-intensity care. However, such associations were not significant among grandfathers.

**Conclusions:**

Our findings highlight the gender differences in depressive symptoms of rural Chinese grandparents caring for grandchildren. Grandparents should be encouraged to engage in grandchild care, but at moderate intensity. The health status of middle- and old-aged adults, particularly females, should be monitored closely. Humanistic care, preventive care and curative treatment strategies focusing on such populations should be developed and refined.

**Supplementary Information:**

The online version contains supplementary material available at 10.1186/s12889-021-11886-3.

## Background

Grandparental childcare is a prevalent form of informal caregiving around the world and one of the main social roles for middle- and old-aged adults [1, 2]. In the West, grandparents usually become caregivers to their own children’s offspring when the adult children are afflicted by any of a number of common problems, including physical or mental illness, drug or alcohol addiction, teen pregnancy, imprisonment, unemployment and full-time work [3–5]. In Europe, grandparents often play a role in childcare when there is limited access to formal childcare institutions, or adult children are affected by commitment to full-time work or by divorce [6]. In Black South African families, looking after young children is more accepted by grandparents because they regard grandparenting as a ‘natural’ responsibility [7]. Similarly, in Asian countries, especially China, grandparents take grandchild care for granted, not only because of common multigenerational family structures and financial supports from adult children (grandchild care is a form of “time-for-money” exchange in some cases), but also as inherited duties derived from traditional and cultural norms [1, 8]. Such customs of multigenerational co-residence and traditional cultural values are validated by the ancient Chinese belief system of Confucianism, which highlights the importance of familial harmony [9] and places great emphasis on the significance of Chinese grandparents’ providing grandchild care.

In the context of the dual Chinese urban-rural social structure, it is easier for rural grandparents to provide custodial grandchild care; urban grandparents are more likely to provide part-time care or supplementary assistance. Despite the rapid urbanization process and unprecedented economic growth have eroded traditional cultural values to some extent, rural residents still observe traditional practices in accordance with inherited norms and beliefs, unlikely many urban residents [10]. Compared with their urban counterparts, Chinese grandparents in rural areas are more likely to be involved in intensive caregiving because of the massive rural-urban migration of the labour force in recent decades [11] and the prevailing preference of intergenerational co-habitation and mutual financial support as the key to ‘family prosperousness’ [12]. Moreover, rural grandparent caregivers are more likely to develop medical problems than their urban peers because of intensive caregiving and a lack of healthcare resources, supporting alternative childcare facilities and other community-based support services or programs [1]. Consequently, we focus on the health of rural Chinese grandparents in this study.

### Grandchild care and grandparents’ depressive symptoms

Depression, a non-communicable disease, is globally prevalent regardless of gaps in economic development [13], and social and cultural factors in various regions [14]. It is evidenced that depression can be a contributory cause of numerous physical health problems, such as inflammation [15], Parkinson disease [16], type-2 diabetes [17] and cardiovascular disease [18], resulting in an increasing burden being placed on individual families and communities, as well as affecting statistics at national level. Moreover, globally, depression is most common in middle- and old-age [13, 19]. As the Chinese population ages rapidly, there is an increasing interest in literature on depressive symptoms of grandparents caring for grandchildren. The literature indicates that caring for grandchildren contributed to reduced depressive symptoms in grandparents in some countries [8, 20, 21], the same scenario is seen in China [22–24]. Based on the theory of role enhancement, multiple social roles lead to improved wellbeing, since individuals gain social integration and gratification from these different areas of social participation [25, 26]. As a type of social role, caring for grandchildren provides grandparents with stronger and more frequent emotional connections with the younger generation and more opportunities for receiving informal and formal support and forging social bonds [27]. According to the existing literature and the theory of role enhancement, we therefore expect that rural Chinese grandparents providing grandchild care have fewer depressive symptoms, compared with those who do not.

### Grandchild-care intensity and grandparents’ depressive symptoms

Despite the evidence that caring for grandchildren relieves grandparents’ depressive symptoms, a few studies still show it to have an overall negative effect [28–30], mainly owing to the added stress involved in caregiving. Role strain theory argues that, when individuals play multiple social roles and undertake a series of social obligations, negative health problems can result when they exceed their physical and psychological capabilities [31, 32]. Following the theory of role strain, the heterogeneous findings in the literature regarding the association between grandchild care and grandparents’ depressive symptoms can be explained by the intensity of grandchild-care involvement. Caring for grandchildren may become a stressful and potentially overwhelming task that can have dire consequences for the physical and psychological health of the individual, particularly for grandparents providing extensive care [33], as they find they have little energy and limited time to maintain other personal social activities or social ties [34]. Studies have shown that high intensity of caregiving is more likely to damage the cognitive health of grandparents [35] and indicate a decline in self-reported health [1]. However, a reduction in care intensity was associated with an increase in life satisfaction in the middle-aged and elderly population in China [36]. Moreover, less intensive grandchild care was demonstrated to have an association with a reduction in depressive symptoms for grandparents in France [37]. Meanwhile increasing grandchild care to an intensive level increased depressive symptoms among grandmothers in Poland, Spain and Sweden [37]. The association between intensity of grandchild care and depressive symptoms of caregivers is seen across Western countries. We hypothesize that moderate-intensity grandchild care is likely to result in fewer depressive symptoms, whereas high-intensity grandchild care is associated with more depressive symptoms for rural grandparents in China.

### Gender context

Despite the well-established association between depressive symptoms and grandparents caring for grandchildren, and multiple influencing factors on caregivers’ depressive symptoms (including demographic characteristics, health status and social participation of the caregiver, grandchildren’s characteristics and living arrangements), most of them did not distinguish between grandmothers and grandfathers. Although important gender differences were found in the grandchild-care experience [38–40] and depressive symptoms were different for males and females [28, 41], most studies analysed grandparents as a demographic group, preventing us from determining a gender differential effect in grandchild-care involvement and depressive symptoms. Given that there are gendered responsibilities and expectations [42], grandmothers usually provide a greater portion of grandchild care than do grandfathers [38, 39, 43]. Empirical evidence on the association between grandchild care and grandparents’ mental health suggests that grandmothers who provide grandchild care are not only at lower risk of depression than those who do not [20], but also experience a higher level of life satisfaction [9, 44] than do grandfathers. Another study using the longitudinal structure of the Survey of Health, Ageing and Retirement in Europe (SHARE) showed a reduction in depressive symptoms in grandmothers who became caregivers [37]. Based on previous evidence around the grandparental role in grandchild care, and depressive symptoms of grandparents caring for grandchildren (considering gender difference), we therefore hypothesize that grandmothers had more depressive symptoms than grandfathers, and grandmothers rather than grandfathers caring for grandchildren are significantly associated with depressive symptoms in rural China.

We attempt to contribute to the literature on grandchild care and depressive symptoms among grandparents by examining the gender differences in depressive symptoms of rural Chinese grandparents caring for grandchildren, using nationally representative data. We propose the following hypotheses: (1) compared with non-caregivers, caregivers are expected to have fewer depressive symptoms; (2) caregivers providing non-intensive of grandchild care are expected to have fewer depressive symptoms, whereas those with intensive grandchild-care involvement are likely to have more depressive symptoms; (3) the above associations are applicable to grandmothers rather than grandfathers; (4) grandmothers suffer more from depressive symptoms than grandfathers.

## Methods

### Data and sample

Data for this study were drawn from the China Health and Retirement Longitudinal Study (CHARLS) conducted in 2015, a nationally representative survey targeting middle- and old-aged adults in China. CHARLS 2015 surveyed 21,789 individuals selected from 450 villages/resident committees in 150 counties/districts in 28 provinces across the country, using a four-stage, stratified, cluster sampling method to select reviewers. The detailed sampling design had been introduced previously [45, 46]. The present study selected grandparents registered as the ‘Agricultural Hukou’ under the Hukou household-registration system [47], with at least one grandchild under 16 years old [27], so as to avoid the possible selection bias suggesting that people without grandchildren are inherently different from those who have grandchildren in terms of health status [48]. Hukou divides Chinese citizens into two categories, with Agricultural Hukou for households in rural areas and Non-agricultural Hukou for those in urban areas [49]. Excluding missing values, the study sample contained 4833 individuals.

### Measurement

**Fig. 1:**
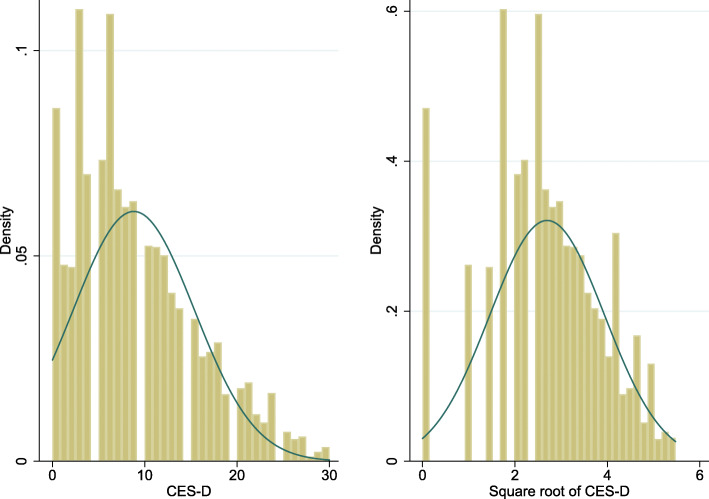
Comparison of distribution of CES-D and its square root

Depressive symptoms served as a dependent variable in this study, measured by the 10-item Center for Epidemiologic Studies Depression Scale (CES-D). The respondents were required to assess their mental and emotional states accurately during the week prior to the interview. Each item used a four-point Likert scale, with an answering category ranging from “Rarely or none of the time (< 1 day)” to “Most or all of the time (5–7 days)”, coding from 0 to 3. The sum score ranged from 0 to 30, with higher values presenting more depressive symptoms. Previous studies suggest that CES-D has good reliability and validity among the Chinese population [50]. According to previous studies that a cut-off point of 12 provides the optimal threshold by which to identify clinically significant depression [51, 52], a score of 12 was also used in this study to generate a binary depression variable (No = 0, Yes = 1). It was used to describe further the prevalence of grandparents’ depression. In order to make the continuous variable CES-D score normally distributed to fit the regression, we transferred the CES-D score to its square root. As Fig. 1 shows, the distribution of its square root was broadly normally distributed, although it skewed slightly left (skewness = − 0.28 and kurtosis = 2.78) [53].

Grandchild care was an independent variable in this study, measured by three indicators. One was grandparents’ self-reported answer to the question, “Did you spend any time taking care of your grandchildren last year”, which was divided into ‘Non-caregivers’ and ‘Caregivers’, a binary variable. The other was the answer to the question, “Approximately how many hours per week did you spend last year on taking care of grandchildren”, which was regarded as a continuous variable (duration) and a categorical variable (no care, low intensity, moderate intensity, high intensity), respectively. Based on previous studies [1, 39, 53, 54], three categories of grandchild-care intensity were identified: low intensity (spending 1–14 h per week), moderate intensity (spending 15–39 h per week) and high intensity (spending ≥40 h per week). The cut-off point of 15 h was referred to in the previous literatures [1, 53], and 40 h was chosen based on standard working time per week (five-day working week and no more than 8 h a day) according to the Labour Law of China and previous studies [55, 56]. The gender of grandparents was used to stratify the full sample into the grandfathers and grandmothers subsamples.

Control variables included age (45–59 years old; ≥60 years old); education level (illiterate; primary school or below; middle school; high school or above); marital status (separated/divorced/widowed/never married; married); work status (unemployed/working); annual household income (poorest/2nd quintile/middle/4th quintile/richest); receiving intergenerational support from children (yes/no); co-habiting with children (yes/no); engaging in social activities (yes/no); suffering from chronic disease (yes/no); ADLs score; IADLs score; and number of grandchildren. Annual household income included individual and other household members’ income (e.g., wages, assets, subsidies, government transfers and other income sources), which was divided into quintiles. Intergenerational support from children included cash and in-kind transfers. Engagement in social activities was defined as any activity participated in between individuals, such as interacting with friends; playing mah-jongg/chess/cards or attending community clubs; providing help for family/friends/neighbours outside the household; attending a sport/social/other club; taking part in community-related organizations; undertaking voluntary or charity work; caring for a sick or disabled adult outside the household; attending an educational or training course; stocking investment and using the internet. We considered using the internet as a type of social activity not only because it is one of the options to this question in the CHALRS questionnaire, but also because prior studies have categorised using the internet as a type of social activity, given that individuals can communicate and form social ties without being affected by limitations on mobility and activity for older adults, and it consequently reduces social isolation, and enhances social integration and support [52, 57, 58]. ADLs score was the sum score of items asking interviewers whether they had difficulties with dressing, bathing or showering, eating, getting into or out of bed, using the toilet, and controlling urination and defecation, with each coding from 0 (no difficulties) to 3 (cannot perform it). IADLs score was measured by totalling the items asking respondents whether they encountered difficulties in performing household tasks, preparing hot meals, shopping for groceries, managing personal finances, making phone calls and taking medications, with the same coding as the former. Detailed definitions and codes of variables are presented in Table 1.

**Table 1:** Definitions/codes of variables

Variables	Definitions/codes
CES-D	Continuous variable, 0–30
Square root of CES-D	Continuous variable, 0–5.48
Grandchild-care duration	Continuous variable, 0–168 h
Grandchild-care provision	No = 0, Yes = 1
Grandchild-care intensity^a^	No care = 0, Low intensity = 1, Moderate intensity = 2, High intensity = 3
Grandchild-care intensity^b^	Low intensity = 0, Moderate intensity = 1, High intensity = 2
Age, years	45–59 years = 1, ≥60 years = 2
Gender	Male = 0, Female = 1
Education level	Illiterate = 1, Primary school or below = 2, Middle school = 3, High school or above = 4
Marital status	Separated/Divorced/Widowed/Never married = 0, Married = 1
Work status	Unemployed = 0, Working = 1
Annual household income	Poorest = 1, 2nd quintile = 2, Middle = 3, 4th quintile = 4, Richest = 5
Receiving intergenerational support from children	No = 0, Yes = 1
Co-habiting with children	No = 0, Yes = 1
Number of grandchildren	Continuous variable
Engagement in social activities	No = 0, Yes = 1
Suffering from chronic disease	No = 0, Yes = 1
ADLs score	Continuous variable, 0–18
IADLs score	Continuous variable, 0–18

*ADLs* Activities of Daily Living, *IADLs* Instrumental Activities of Daily Living

^a^Among all participates

^b^Among the caregivers

### Coarsened exact matching method

Evidence shows that both matched sampling and regression adjustment can be expected to reduce bias [59]. Matching method application is more robust than regression analysis alone [60]. Initially, we used the Coarsened exact matching (CEM) method put forward by Iacus et al. [61, 62] to balance the multidimensional distribution of covariates between the two compared groups (non-caregivers and caregivers), and thereby reduce the explanatory variable’s degree of dependence on the estimation model and further decrease the biases. CEM is a matching method of the class Monotonic Imbalance Bounding (MIB), which shows the basic advantage over other matching methods that the bound on balance for one covariate can be studied and improved in isolation, as this won’t affect any other covariates chosen for balancing [62, 63]. It is preferable to other matching procedures (e.g., propensity score matching, PSM) in terms of processing more efficiently and reducing model dependence, estimation error, variance and bias [64]. It does not require further conduct balance checking or restrict data to common support, as is required by PSM.

The CEM algorithm consists of three principal procedures [61]. Firstly, each variable is coarsened by recoding, and thereby indistinguishable values are grouped and allotted the same numerical value (groups may have the same or different sizes). Secondly, the coarsened data are matched using an “exact matching” algorithm, and unmatched units are pruned. Thirdly, the coarsened data are removed and the uncoarsened (original) values of the matched data are retained. Additionally, a CEM-weights variable is generated to equalize the number of observations within comparison groups, where unmatched units are 0 and matched units are larger than 0 but less than 1 [61]. For balance checking of two compared groups, multivariate imbalance measure L_1_ is employed, of which size depends on the dataset and the variables selected. L_1_ ranges from 0 to 1, where 0 and 1 represent perfect global balance and complete separation, respectively, and a larger value indicates greater imbalance between two groups. A good match usually reduces the value of L_1_ [65]. The L_1_ statistic is calculated as follows [66]. Firstly, the covariates are coarsened into bins. Then, the discretized variables are cross-tabulated as X_1_× … … × X_k_ for the treated and control groups, respectively, and k-dimensional relative frequencies are recorded for the treated ${\mathcal{f}}_{\ell 1\dots \mathit{\ell k}}$ and the control *ℊ*_*ℓ*1. . *ℓk*_ units. Finally, the measure of imbalance is the absolute difference over all the cell values:

$$ {\mathrm{L}}_1\left(\mathcal{f},\mathcal{g}\right)=\frac{1}{2}\sum \limits_{\ell 1\dots \mathit{\ell k}}\mid {\mathcal{f}}_{\ell 1\dots \mathit{\ell k}}-{\mathcal{g}}_{\ell 1..\mathit{\ell k}}\mid $$

In the current study, we matched the socioeconomic characteristics and variables related to grandchild care according to the literature on CEM progress [53, 67], including age, education level, marital status, work status, annual household income, whether receiving intergenerational support from children, whether co-habiting with children, and number of grandchildren.

### The moderation effect and stratified analysis

We first examined the moderation effect of gender on the association between grandchild care and depressive symptoms by creating interaction terms in multilevel linear regression. The predictive margins and the average marginal effects were presented to interpret the gender differences visually. Furthermore, taking gender differences in life expectancy (e.g., females generally live longer than males), socioeconomic status (e.g., males have higher educational attainment and better financial resources than females), and labour market (e.g., males have longer employment history than females) into account, we maintain that they are also likely to contribute to the gender gaps in the association between grandchild care and depressive symptoms. We therefore stratified the analyses by gender and further explored the possible gender differences in such an association.

### Statistical analysis

The Chi-square test for categorical variables and Univariate ANOVA for continuous variables were used to compare caregivers and non-caregivers in the unmatched and matched cohorts. Matched weights were considered in all analyses in matched groups. Since the data of this study were drawn from CHARLS, with a four-stage stratified cluster sampling, the dependence among observations could exist on several levels of the hierarchy. To remove the cluster effect of observations at different levels of the data hierarchy [68], we fitted four-level multiple linear regression models (individual at level 1; nested within the community at level 2; nested within the city at level 3; nested within the province at level 4). An Intra-class Correlation Coefficient (ICC) is used to check the applicability and validity of the multilevel model. The ICC measures correlation among observations within a cluster, ranging from 0 to 1. A multilevel regression model is appropriate for the analysis when ICC is greater than 0 [68, 69]. In this study, the ICC values were 0.038, 0.071 and 0.113 in the grandfather subsample; and 0.023, 0.060 and 0.112 in the grandmother subsample, at province, city and community levels, respectively, which made multilevel linear regression suitable.

Subsequently, we examined the association between grandchild-care duration and depressive symptoms among all participants (including caregivers and non-caregivers) in Model 1; the association between grandchild-care provision and depressive symptoms among all participants in Model 2; and the association between grandchild-care intensity and depressive symptoms, respectively, among all participants in Model 3 and caregivers in Model 4, by using multilevel linear regression, controlling for grandparents’ socioeconomic characteristics (including age, education level, marital status, work status, annual household income) and health status (including chronic disease, ADLs, IADLs), receiving intergenerational support from children, co-habiting with children, number of grandchildren and level of social engagement. Stata statistical software (version 15.0; StataCorp LP, College Station, Texas) was used for all analyses.

## Results

Following the matching using CEM, 1975 non-caregivers and 2212 caregivers were identified for further analysis (*N* = 4187). The multivariate imbalance measure L_1_ was improved from 0.500 to nearly zero, and all variables matched were also close to zero, which indicated a good matching performance. Table 2 presents the basic characteristics of non-caregivers and caregivers before and after matching. It was clear that there were significant differences in most characteristics of non-caregivers and caregivers before matching. No statistical difference was found in any characteristics of non-caregivers and caregivers after the matching (*P* > 0.10), which further indicated that non-caregivers and caregivers were more comparable and balanced.

**Table 2:** Basic characteristics of non-caregivers and caregivers in unmatched and matched cohorts

Variables	Unmatched (*N* = 4833)			Matched (*N* = 4187)		
	Non- caregivers (%)	Caregivers (%)	*P*-value	Non- caregivers (%)	Caregivers (%)	*P*-value^c^
N	2199	2634		1975	2212	
Age^a^, years			< 0.001			0.993
45–59	711 (32.33)	1223 (46.43)		881 (44.62)	987 (44.62)	
≥60	1488 (67.67)	1411 (53.57)		1094 (55.38)	1225 (55.38)	
Education level^a^			< 0.001			1.000
Illiterate	650 (29.56)	663 (25.17)		479 (24.23)	536 (24.23)	
Primary school or below	1069 (48.61)	1203 (45.67)		967 (48.96)	1083 (48.96)	
Middle school	361 (16.42)	560 (21.26)		407 (20.61)	456 (20.61)	
High school or above	119 (5.41)	208 (7.90)		122 (6.19)	137 (6.19)	
Marital status^a^			< 0.001			0.983
Married	582 (26.47)	411 (15.60)		254 (12.84)	284 (12.84)	
Separated/Divorced/Wido-wed/Never married	1617 (73.53)	2223 (84.40)		1721 (87.16)	1928 (87.16)	
Work status^a^			< 0.001			0.994
Unemployed	712 (32.38)	690 (26.20)		460 (23.28)	515 (23.28)	
Working	1487 (67.62)	1944 (73.80)		1515 (76.72)	1697 (76.72)	
Annual household income^a^			< 0.001			0.999
Poorest	203.28 (172.07)	219.23 (183.78)		212.12 (173.27)	220.90 (183.18)	
2nd quintile	1225.98 (446.14)	1198.56 (442.78)		1304.82 (467.74)	1224.17 (462.97)	
Middle	4622.72 (1928.62)	4687.21 (1964.16)		4740.37 (1886.45)	4786.37 (1959.94)	
4th quintile	17,909.42 (6331.63)	18,762.86 (6572.85)		17,893.18 (6046.25)	18,599.94 (6400.95)	
Richest	12,2600.7 (414,961.1)	101,823.9 (184,383.4)		114,110.5 (336,610.3)	101,205.1 (187,140.5)	
Receiving intergenerational support from children^a^			0.814			0.982
No	312 (14.19)	380 (14.43)		221 (11.21)	248 (11.21)	
Yes	1887 (85.81)	2254 (85.57)		1754 (88.79)	1964 (88.79)	
Co-habiting with children^a^			< 0.001			1.000
No	1514 (68.85)	1468 (55.73)		1150 (58.23)	1288 (58.23)	
Yes	685 (31.15)	1166 (44.27)		825 (41.77)	924 (41.77)	
Number of grandchildren^b^	2.65 (1.89)	3.00 (1.97)	< 0.001	2.55 (1.64)	2.55 (1.64)	1.000
Engagement in social activities^a^			0.001			0.085
No	1120 (50.93)	1211 (45.98)		958 (48.50)	1014 (45.84)	
Yes	1079 (49.07)	1423 (54.02)		1017 (51.50)	1198 (54.16)	
Suffering from chronic disease^a^			0.048			0.923
No	579 (26.33)	761 (28.89)		575 (29.11)	647 (29.25)	
Yes	1620 (73.67)	1873 (71.11)		1400 (70.89)	1565 (70.75)	
ADLs score^b^	0.69 (1.77)	0.50 (1.33)	< 0.001	0.55 (1.44)	0.50 (1.33)	0.553
IADLs score^b^	1.34 (2.78)	1.03 (2.32)	< 0.001	1.09 (2.41)	1.02 (2.35)	0.113

*ADLs* Activities of Daily Living, *IADLs* Instrumental Activities of Daily Living

^a^Chi-square test

^b^Univariate ANOVA

^c^Considering match weights

Overall, 52.83% of grandparents played a role in grandchild care, with an average of 36.00 (SD = 52.35) hours per week. Grandmothers were more likely to provide grandchild care (54.42% vs 51.43%) with longer duration (39.24 h vs 33.15 h), and they gave more care at high intensity (61.46% vs 51.01%) than grandfathers.

Table 3 shows depressive symptoms of all participates, non-caregivers and caregivers, as well as those of caregivers with various levels of intensity across genders. Gender differences in depressive symptoms were found in each comparison group (*P* < 0.001), indicating that grandmothers had more depressive symptoms than grandfathers.

**Table 3:** Comparison of depressive symptoms across gender in matched cohorts

Variables	Grandchild-care duration	Grandchild-care provision		Grandchild-care intensity		
	Mean (SD)	Non-caregivers	Caregivers	Low	Moderate	High
		Mean (SD)	Mean (SD)	Mean (SD)	Mean (SD)	Mean (SD)
Gender						
Male	7.55	7.82	7.32	7.15	7.34	7.35
	(6.06)	(6.14)	(5.98)	(5.81)	(5.97)	(6.04)
Female	10.23	10.62	9.91	9.90	9.39	10.08
	(6.92)	(7.10)	(6.75)	(7.07)	(6.48)	(6.73)
*P*-value	< 0.001	< 0.001	< 0.001	< 0.001	< 0.001	< 0.001

Depressive symptoms were measured by CES-D

Univariate ANOVA was employed

**Fig. 2:**
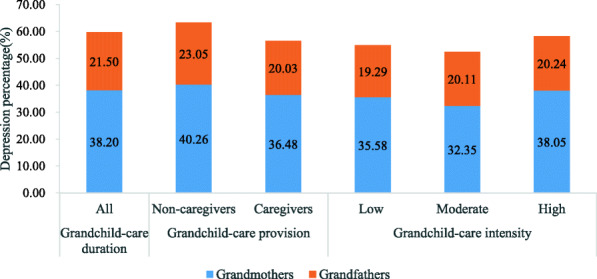
Depression prevalence of grandfathers and grandmothers caring for grandchildren in matched cohorts

Figure 2 further presents depression prevalence of grandfathers and grandmothers caring for grandchildren. We found that grandmothers suffered from higher risks of depression than grandfathers (38.20% vs 21.50%). Caregivers had lower depression prevalence compared with non-caregiver counterparts (20.03% vs 23.50% among grandfathers, and 36.48% vs 40.26% among grandmothers). The more intensive the level of care that grandfathers provided, the higher the risk of depression (19.29% vs 20.11% vs 20.24%). Grandmothers had the lowest risk of depression when participating in caregiving at moderate intensity (32.35%).

**Fig. 3:**
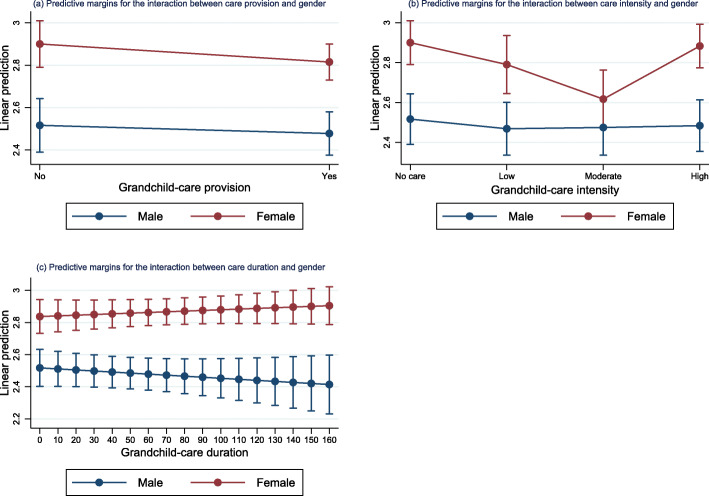
Predictive margins for the interaction between gender and grandchild care in matched cohorts

**Fig. 4:**
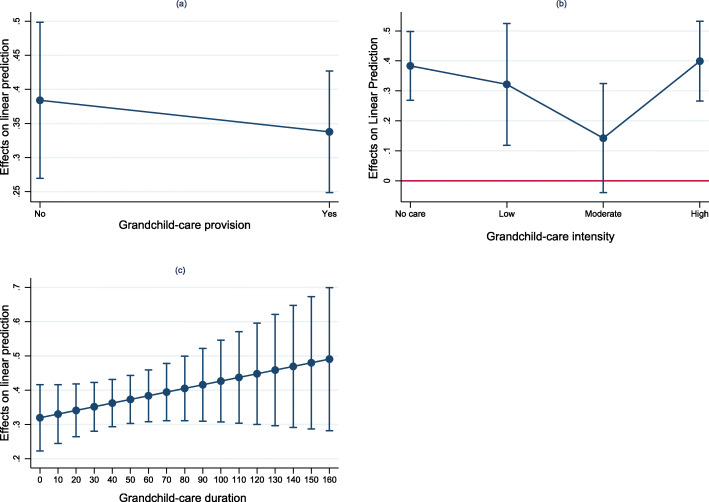
Average marginal effects of gender in matched cohorts

Figure 3 presents the adjusted prediction margins for the interaction between gender and grandchild-care provision, intensity and duration. Gender played a moderating role on the association between care intensity and depressive symptoms. The negative interaction effect between moderate-intensity care and female suggested that grandmothers providing care at moderate intensity suffered fewer from depressive symptoms. Detailed results for the multilevel linear regression examining the moderation effect are reported in Table S1. Figure 4 shows the average marginal effect of gender. The findings that the average marginal effects of gender were significantly greater than zero indicated that grandmothers had more depressive symptoms than grandfathers, except for those with moderate-intensity care. The gender difference diminished when grandchild care-intensity increased from low to moderate. However, greater gender difference was found when intensity reached high level (see Fig. 4b).

Table 4 further shows the gender differences in depressive symptoms of grandparents caring for grandchildren, based on the grandfathers and grandmothers subsamples and using multilevel linear regression. We found that depressive symptoms were not significantly associated with grandchild-care duration (see Model 1), whereas they were correlated with care intensity (see Models 2, 3 and 4). Models 2 and 3 indicate that grandmothers providing grandchild care, particularly at moderate intensity, were associated with fewer depressive symptoms than non-caregivers (Coef. = − 0.087, 95%CI: − 0.163, − 0.010; Coef. = − 0.291, 95%CI: − 0.435, − 0.147). Model 4 indicates that, compared with those with low care intensity, grandmothers with moderate intensity of grandchild care were associated with fewer depressive symptoms (Coef. = − 0.171, 95%CI: − 0.313, − 0.029), but those with high intensity of grandchild care were associated with more depressive symptoms, although the difference was not significant (Coef. = 0.110, 95%CI: − 0.184, 0.182). However, the above associations were not significant among grandfathers.

**Table 4:** Gender differences in depressive symptoms of grandparents caring for grandchildren in matched cohorts

Variables	Grandfathers				Grandmothers			
	Model 1	Model 2	Model 3	Model 4	Model 1	Model 2	Model 3	Model 4
	Coef.	Coef.	Coef.	Coef.	Coef.	Coef.	Coef.	Coef.
	(95%CI)	(95%CI)	(95%CI)	(95%CI)	(95%CI)	(95%CI)	(95%CI)	(95%CI)
Fixed effects								
Grandchild-care duration, hours	− 0.0006				0.0004			
	(− 0.002–0.001)				(− 0.001–0.001)			
Grandchild-care provision (Ref: No)								
Yes		−0.034				−0.087^*^		
		(− 0.150–0.083)				(− 0.163 - -0.010)		
Grandchild-care intensity (Ref: No care)								
Low			− 0.047				− 0.112	
			(− 0.196–0.102)				(− 0.257–0.032)	
Moderate			− 0.031				−0.291^***^	
			(− 0.173–0.111)				(− 0.435 - -0.147)	
High			−0.031				− 0.017	
			(− 0.174–0.113)				(−0.120–0.085)	
Grandchild-care intensity (Ref: Low)								
Moderate				−0.004				−0.171^*^
				(−0.190–0.182)				(−0.313 - -0.029)
High				−0.001				0.110
				(−0.184–0.182)				(−0.064–0.284)
Age, years (Ref: 45-49)								
≥ 60	0.088	0.087	0.087	0.201^*^	−0.139^*^	− 0.142^*^	−0.133^*^	− 0.176^*^
	(−0.054–0.229)	(−0.055–0.229)	(−0.055–0.229)	(0.021–0.381)	(−0.253 - -0.026)	(− 0.252 - -0.032)	(− 0.244 - -0.023)	(−0.312 - -0.040)
Education level (Ref: Illiterate)								
Primary school or below	0.176^†^	0.176^†^	0.176^†^	0.115	−0.031	− 0.029	−0.030	− 0.032
	(−0.024–0.376)	(−0.023–0.375)	(−0.023–0.374)	(−0.063–0.292)	(−0.173–0.111)	(−0.170–0.112)	(−0.171–0.111)	(−0.154–0.091)
Middle school	0.001	0.001	3.20e-05	0.006	−0.228^*^	−0.222^*^	− 0.230^*^	−0.255^**^
	(−0.229–0.230)	(−0.228–0.229)	(−0.228–0.228)	(−0.221–0.233)	(−0.419 - -0.037)	(− 0.412 - -0.032)	(− 0.419 - -0.042)	(−0.441 - -0.070)
High school or above	−0.261	−0.265	− 0.266	−0.049	− 0.314	−0.321	− 0.331	−0.590^*^
	(−0.645–0.122)	(−0.647–0.117)	(−0.647–0.115)	(−0.310–0.212)	(−1.077–0.448)	(−1.063–0.422)	(−1.074–0.412)	(−1.110 - -0.069)
Marital status (Ref: Separated/Divorced/Widowed/Never married)								
Married	−0.068	− 0.071	−0.071	− 0.128	−0.131	− 0.130	−0.133	− 0.178
	(−0.233–0.098)	(−0.232–0.090)	(−0.232–0.090)	(−0.396–0.140)	(−0.299–0.038)	(−0.298–0.039)	(−0.304–0.039)	(−0.402–0.045)
Work status (Ref: Unemployed)								
Working	−0.034	− 0.033	−0.032	0.010	0.059	0.052	0.057	0.075
	(−0.184–0.117)	(−0.182–0.116)	(−0.183–0.118)	(−0.176–0.196)	(−0.106–0.224)	(−0.116–0.220)	(−0.107–0.222)	(−0.120–0.271)
Annual household income (Ref: Poorest)								
2nd quintile	0.009	0.007	0.006	0.006	0.078	0.076	0.076	0.101
	(−0.197–0.216)	(−0.197–0.210)	(−0.199–0.211)	(−0.226–0.238)	(−0.045–0.201)	(−0.048–0.200)	(−0.049–0.201)	(−0.078–0.280)
Middle	−0.034	− 0.035	−0.036	− 0.061	−0.036	− 0.036	−0.030	0.034
	(−0.186–0.118)	(−0.187–0.117)	(−0.188–0.117)	(−0.293–0.171)	(−0.229–0.157)	(−0.231–0.159)	(−0.224–0.164)	(−0.207–0.274)
4th quintile	−0.016	− 0.018	−0.018	− 0.115	−0.014	− 0.016	−0.022	− 0.154
	(−0.216–0.183)	(−0.214–0.179)	(−0.213–0.178)	(−0.352–0.123)	(−0.190–0.163)	(−0.192–0.160)	(−0.197–0.153)	(−0.368–0.061)
Richest	−0.185^*^	− 0.186^*^	−0.186^*^	− 0.280^*^	−0.238^*^	− 0.240^*^	−0.237^*^	− 0.282^*^
	(−0.348 - -0.022)	(− 0.349 - -0.023)	(− 0.348 - -0.024)	(−0.493 - -0.066)	(− 0.463 - -0.013)	(− 0.462 - -0.017)	(−0.457 - -0.018)	(− 0.507 - -0.057)
Receiving intergenerational support from children (Ref: No)								
Yes	0.020	0.020	0.020	−0.028	0.027	0.028	0.029	−0.066
	(−0.197–0.237)	(−0.197–0.237)	(−0.197–0.238)	(−0.200–0.145)	(−0.229–0.282)	(−0.225–0.281)	(−0.229–0.286)	(−0.329–0.196)
Co-habiting with children (Ref: No)								
Yes	−0.023	− 0.023	−0.023	− 0.017	−0.015	− 0.012	−0.009	− 0.092
	(−0.136–0.090)	(−0.136–0.091)	(−0.136–0.090)	(−0.175–0.140)	(−0.200–0.169)	(−0.196–0.171)	(−0.190–0.172)	(−0.273–0.089)
Number of grandchildren	0.012	0.011	0.011	−0.008	0.015	0.018	0.017	0.038^†^
	(−0.018–0.043)	(−0.019–0.041)	(−0.019–0.041)	(−0.054–0.037)	(−0.034–0.063)	(−0.033–0.068)	(−0.033–0.067)	(−0.003–0.080)
Engagement in social activities (Ref: No)								
Yes	−0.062	− 0.062	−0.062	− 0.210^***^	−0.103^*^	− 0.098^*^	−0.089^†^	− 0.080
	(−0.174–0.050)	(−0.175–0.051)	(−0.174–0.051)	(−0.299 - -0.120)	(− 0.202 - -0.004)	(− 0.195 - -0.001)	(−0.187–0.009)	(−0.196–0.037)
Suffering from chronic disease (Ref: No)								
Yes	0.198^**^	0.199^**^	0.199^**^	0.175^*^	0.294^***^	0.295^***^	0.293^***^	0.239^***^
	(0.049–0.347)	(0.052–0.347)	(0.052–0.347)	(0.006–0.344)	(0.156–0.433)	(0.157–0.432)	(0.156–0.431)	(0.126–0.352)
ADLs score	0.127^***^	0.127^***^	0.127^***^	0.159^***^	0.108^***^	0.107^***^	0.109^***^	0.110^***^
	(0.073–0.181)	(0.073–0.181)	(0.073–0.181)	(0.096–0.222)	(0.066–0.151)	(0.064–0.150)	(0.066–0.152)	(0.052–0.168)
IADLs score	0.059^***^	0.059^***^	0.059^***^	0.063^***^	0.090^***^	0.091^***^	0.091^***^	0.092^***^
	(0.030–0.088)	(0.030–0.088)	(0.030–0.089)	(0.031–0.096)	(0.064–0.117)	(0.064–0.117)	(0.064–0.119)	(0.065–0.119)
Random-effects (intercept)								
Level 4 (SE)	0.031	0.031	0.031	0.022	0.015	0.016	0.016	0.001
	(0.013)	(0.013)	(0.013)	(0.014)	(0.013)	(0.013)	(0.013)	(0.009)
Level 3 (SE)	0.042	0.043	0.043	0.042	0.036	0.036	0.035	0.049
	(0.016)	(0.016)	(0.016)	(0.033)	(0.015)	(0.015)	(0.015)	(0.031)
Level 2 (SE)	0.034	0.033	0.033	0.005	0.064	0.061	0.063	0.006
	(0.020)	(0.020)	(0.020)	(0.006)	(0.027)	(0.026)	(0.027)	(0.025)

Model 1 examined the association between grandchild-care duration and depressive symptoms among all participants. Model 2 examined the association between grandchild-care provision and depressive symptoms among all participants. Models 3 and 4 examined the association between grandchild-care intensity and depressive symptoms, among all participants and caregivers, respectively

Multilevel linear regression was employed

Depressive symptoms were measured by square root of CES-D

*Ref* Reference, *SE* Standard Error, *CI* Confidence Interval, *ADLs* Activities of Daily Living, *IADLs* Instrumental Activities of Daily Living

^***^ *p* < 0.001, ^**^ *p* < 0.01, ^*^ *p* < 0.05, ^†^*p* < 0.1

## Discussion

In this study, we examine the gender differences in depressive symptoms of rural Chinese grandparents caring for grandchildren using nationally representative data. Consistent with hypothesis (1), grandmothers providing grandchild care were associated with fewer depressive symptoms compared with non-caregivers. Partly consistent with hypothesis (2), grandmothers with moderate intensity of grandchild care were associated with fewer depressive symptoms than those with low-intensity care. However, it was not strongly evidenced that grandmothers giving high-intensity care were likely to have more depressive symptoms. Consistent with hypothesis (3), the association between grandchild care and depressive symptoms was not significant among grandfathers. Moreover, grandmothers undertook more grandchild care and suffered more from depressive symptoms than did grandfathers.

As a consequence of increasing life expectancy, improved health of the elderly, and higher levels of divorce among modern adults, grandparents are becoming increasingly actively involved in family life around the world [43, 70]. They play significant social roles in caring for grandchildren [53]. In a study of Israel and 17 European countries, nearly half of grandparents provided grandchild care [37]. In the US, 25% of children under the age of 5 have been cared for by grandparents [71]. The current study showed that over half of rural Chinese grandparents spent an average of 36.00 h per week taking care of grandchildren, much higher than Taiwanese (20.3%) [8] and Korean grandparents (4.8%) [54], as well as Chinese American (an average of 11.96 h a week) [27] and Spanish grandparents (an average of 23 h a week) [43]. In rural China, grandparents usually assume the duty of grandchild care to alleviate the burden on their adult children, particularly if the latter are employed [11, 35], since adult children often migrate from rural to urban locations to seek better employment opportunities and are obliged to leave their children with grandparents [36].

Grandmothers provided more hours of grandchild care than did grandfathers, in line with their Spanish peers [43]. They were also more likely to give grandchild care at high intensity than were grandfathers, similar to recent studies in China [9] and elsewhere in Europe [37]. This obvious gender-based gap presents an disparity of care assignment in rural Chinese society [72]. Females traditionally hold more responsibilities and obligations as ‘kin keepers’ [73]: they not only play with their grandchildren and take them on excursions, but also perform feeding, changing clothing/nappies, washing clothes and bathing with greater frequency than do grandfathers. Grandfathers typically play roles centred around entertainment and companionship [74, 75].

Moreover, grandmothers reported more depressive symptoms than grandfathers, and such differences increased when caregivers were involved in high intensity of care. A great number of studies have confirmed that females suffer more frequently from depressive symptoms [43, 76, 77]. Possible explanations for this are gender differences in terms of family/social involvement; feelings of responsibility in family matters; socioeconomic characteristics (e.g., education, income and marriage); social factors (e.g., social role, life events, social ties and social support); and psychological factors (e.g., vulnerability, mastery) [78, 79]. When increasing care to intensive level, the above gender differences may become greater and lead to increased gaps in depressive symptoms. Therefore, we further stratified the analyses by gender so as to take gender differences in such aspects into account. We found a significant association between grandchild care and depressive symptoms among grandmothers, but not in grandfathers.

Grandmothers providing grandchild care, particularly at moderate intensity, had fewer depressive symptoms, consistent with a study in rural China [24]. This confirms the theory of role enhancement that grandparents gain significant psychosocial benefits from involvement in caregiving [67]. Grandparents’ psychosocial benefits are mainly derived from emotional fulfillment rewards through participating in interactive intergenerational activities; learning opportunity rewards that come from having access to the use of mobile phones and the internet to maintain better contact with family and friends; and relation-oriented rewards in terms of increased self-esteem and self-confidence [80]. Moreover, grandchild care provides grandparents with new purpose in later life, reinforces bonds between family members, and enhances family happiness [29, 81, 82]. These positive events contribute to their being at lower risk of more depressive symptoms.

However, it is worth noting that high intensity of care was associated with more depressive symptoms among grandmothers, compared with those with low-intensity care, although this was not significant. Such possible association might be explained by the theory of role strain that mental health may be damaged if obligations of grandchild care exceed grandparents’ physical and psychological resources (e.g., educational attainment, income and mental and physical health) [31]. High intensity of grandchild care usually leads to increased stress as a result of time pressure, exhaustion and loss of sleep [83]. It also results in strained relationships with spouses or children [84] and sacrifice of self-interest/personal wellbeing [85]. The exertion and pressure associated with confronting and solving the problems that naturally arise in the course of grandchild care will eventually exact a toll on the health of the caregiver. Limiting grandparental involvement to moderate caregiving is beneficial to mental health, including alleviating depressive symptoms [1, 67]. Grandparents providing grandchild care should pay attention to the degree of care intensity and try to prevent care duties becoming a serious burden on them. However, whether excessive intensity of care is significantly associated with increased depressive symptoms, and the possible causal mechanisms for this are needed to be further examined in future studies.

With regard to the above associations applied to grandmothers rather than grandfathers, it is in line with a study in 10 European countries [53]. This further suggests the gender differences in depressive symptoms of grandparents looking after grandchildren. Previous empirical studies have found that grandmothers who look after grandchildren had a lower risk of developing depression and attributed this to males’ more detached role in childrearing and care [20, 30]. This may be particularly important in Chinese society, given strongly differentiated gender roles and responsibilities. The gendered difference in grandchild-care involvement suggests that grandfathers may acquire fewer psychological resources and benefits, but also suffer less from stress and other psychological burdens in later life because of their limited involvement in caring for grandchildren [86, 87]. With regard to grandmothers, the increased contact with adult children during grandchild care is usually a reinforcement of the mother-child relationship and might work as another mechanism for reducing depressive symptoms. Heavy grandchild-care involvement and probable strained relationships deriving from overly frequent contact with adult children are more likely to increase role strain for grandmothers than grandfathers and, therefore, could translate into a deeper health deficit (e.g., an increase in depressive symptoms). However, further investigation, including qualitative studies, might help to determine the mechanism of the gender differences in the association between grandparental childcare and depressive symptoms.

The current study supplements and improves upon the existing literature on grandchild care and depressive symptoms among grandparents in rural China. Unlike previous studies, we employ a matching method to balance the multidimensional distribution of covariates between non-caregivers and caregivers, and thereby reduce the degree of grandchild-care dependence on estimation models and further reduce biases. Moreover, we use large-scale nationally representative data from the largest developing country (China), as compared with most previous studies in developed countries. Furthermore, we employ multilevel regression to remove the cluster effect of individuals at different levels of hierarchy in accordance with the sampling method.

### Limitations

We acknowledge several limitations of the current study and, therefore, the findings should be interpreted with caution. Firstly, owing to the limited data on characteristics of grandchildren in CHARLS, we took only the number of grandchildren into consideration. Detailed characteristics of grandchildren (age, gender, health status and so on) cannot be extracted from the questionnaire, which may make estimation less precise. Secondly, we have no information regarding the experiences and assignments of grandparent caregivers. No data are available on their reasons for assuming the responsibility for grandchild care (e.g., out of a sense of obligation); the caregiving pattern (e.g., temporary or primary caregiver, custodial or non-custodial caregiver, sole caregiver or caregiver with other helpers); their feelings during care involvement; the daily matters and activities they perform; the quality of caregiving; or their relationships with their adult children. Future studies covering such information may contribute to identifying the causal pathways underlying the association between grandparental childcare and depressive symptoms, as well as gender differences. Thirdly, grandparents’ self-reported caregiving hours can be subject to recall bias and social-desirability bias [9]. Fourthly, in view of past studies indicating that grandparents in rural China are more likely to be involved in intensive caregiving [11, 36], and their potential medical problems owing to a lack of healthcare resources and supporting alternative childcare facilities compared with urban areas [1], we focused on a rural sample in this study. The possible rural-urban gender differences in depressive symptoms of grandparents caring for grandchildren are needed to be identified. Such a study would contribute to detecting potential gender disparities in grandchild-care engagement and health conditions between rural and urban grandparents, and may be used to further improve policy formulation. Fifthly, although we found there to be an association between grandchild care and depressive symptoms among grandmothers rather than grandfathers, further investigation, including qualitative and quantitative studies, is needed to identify the causal mechanisms, and to assess the extent to which the observed gender differences depend on grandmothers’ and grandfathers’ differential roles, expectations and experiences.

In addition, we declined to use the longitudinal dataset for CHARLS to examine the gender differences in the causal relationship between grandchild care and depressive symptoms among grandparents, owing to limited grandchild information. In order to reduce biased estimation as far as possible, the present study examined only the association between grandchild care and depressive symptoms, although CEM, a quasi-experimental matching method for causal inference, was employed [88]. Based on the findings of this study, it is necessary to examine the causal relationship between grandparental caregiving patterns and depressive symptoms, as well as the gender differences, by using other, more detailed, precise and comprehensive longitudinal data related to grandchild care.

## Conclusion

The present study highlights the gender differences in depressive symptoms of rural Chinese grandparents caring for grandchildren. Grandmothers suffered more from depressive symptoms than grandfathers. Grandmothers with grandchild provision, particularly at moderate intensity, were associated with fewer depressive symptoms compared with non-caregivers. We recommend that grandparents be encouraged to engage in grandchild care, but at moderate intensity. Against the background of an aging population and a potentially increasing birth rate with the implementation of the universal two-child policy in China [89], more and more elderly citizens will become involved in grandchild care in future. The mental and physical health of middle- and old-aged adults—and, in particular, females—should be monitored closely. Humanistic care, preventive care and curative treatment strategies focusing on middle- and old-aged females should, therefore, be developed and refined.

## Supplementary Information

**Additional file 1: Table S1.** The moderation effect of gender on the association between grandchild care and depressive symptoms in matched cohort.

## Data Availability

The data that support the findings of this study are openly available in CHARLS website: http://charls.pku.edu.cn.

## Abbreviations

SHARE: Survey of Health, Ageing and Retirement in Europe
CHARLS: China Health and Retirement Longitudinal Study
CES-D: Center for Epidemiologic Studies Depression Scale
CEM: Coarsened exact matching
MIB: Monotonic Imbalance Bounding
PSM: Propensity score matching
ICC: Intra-class Correlation Coefficient
