# Supplementary material for: Gender differences in depressive symptoms of rural Chinese grandparents caring for grandchildren
Source: BMC Public Health. 2021 Oct 11;21:1838. doi: 10.1186/s12889-021-11886-3 (PMC8507248; doi:10.1186/s12889-021-11886-3)
Supplement: Supplementary file 1 — Additional file 1: Table S1. The moderation effect of gender on the association between grandchild care and depressive symptoms in matched cohort. [file 12889_2021_11886_MOESM1_ESM.docx]

Table S1 The moderation effect of gender on the association between grandchild care and depressive symptoms in matched cohort

| Variables | Model 1 | Model 2 | Model 3 | Model 4 |
| --- | --- | --- | --- | --- |
|  | Coef. | Coef. | Coef. | Coef. |
|  | (95%CI) | (95%CI) | (95%CI) | (95%CI) |
| Grandchild-care duration×Female | 0.001 |  |  |  |
|  | (-0.001 - 0.003) |  |  |  |
| Grandchild-care provision×Female |  | -0.046 |  |  |
|  |  | (-0.195 - 0.102) |  |  |
| Low intensity×Female |  |  | -0.062 |  |
|  |  |  | (-0.296 - 0.172) |  |
| Moderate intensity×Female |  |  | -0.241^***^ | -0.157 |
|  |  |  | (-0.415 - -0.068) | (-0.422 - 0.108) |
| High intensity×Female |  |  | 0.016 | 0.101 |
|  |  |  | (-0.180 - 0.211) | (-0.171 - 0.373) |
| Gender (Ref: Male) | 0.319^***^ | 0.384^***^ | 0.383^***^ | 0.300^***^ |
| Female | (0.223 - 0.416) | (0.270 - 0.498) | (0.269 - 0.498) | (0.091 - 0.509) |
|  |  |  |  |  |
| Grandchild-care duration, hours | -0.001 |  |  |  |
|  | (-0.002 - 0.001) |  |  |  |
| Grandchild-care provision (Ref: No) |  |  |  |  |
| Yes |  | -0.039 |  |  |
|  |  | (-0.153 - 0.076) |  |  |
| Grandchild-care intensity (Ref: No care) |  |  |  |  |
| Low |  |  | -0.048 |  |
|  |  |  | (-0.198 - 0.102) |  |
| Moderate |  |  | -0.042 |  |
|  |  |  | (-0.182 - 0.098) |  |
| High |  |  | -0.033 |  |
|  |  |  | (-0.173 - 0.107) |  |
| Grandchild-care intensity (Ref: Low) |  |  |  |  |
| Moderate |  |  |  | -0.009 |
|  |  |  |  | (-0.185 - 0.168) |
| High |  |  |  | 0.008 |
|  |  |  |  | (-0.178 - 0.194) |

*Note:* Multilevel linear regression was conducted, controlling grandparent’s socioeconomic characteristics (including age, education level, marital status, work status, annual household income) and health status (including chronic disease, ADL, IADL), receipting intergenerational support from children, co-residence with children, number of grandchildren and social engagement.

Model 1 examined the association between grandchild-care duration and depressive symptoms among all participants. Model 2 examined the association between grandchild-care provision and depressive symptoms among all participants. Models 3 and 4 examined the association between grandchild-care intensity and depressive symptoms, among all participants and caregivers, respectively.

Depressive symptoms were measured by square root of CES-D.

Ref = Reference; CI = Confidence interval.

^***^ p<0.001, ^**^ p<0.01, ^*^ p<0.05, ^†^p<0.1
